# Renal effects of *Mammea africana* Sabine (Guttiferae) stem bark methanol/methylene chloride extract on L-NAME hypertensive rats

**DOI:** 10.4103/0253-7613.68418

**Published:** 2010-08

**Authors:** Elvine Pami Nguelefack-Mbuyo, Théophile Dimo, Télesphore Benoit Nguelefack, Alain Bertrand Dongmo, Pierre Kamtchouing, Albert Kamanyi

**Affiliations:** 1 Laboratory of Animal Physiology, Department of Animal Biology and Physiology, University of Yaounde I, P.O.Box 812 Yaounde, Cameroon; 2 Laboratory of Animal Physiology and Phytopharmacology, Department of Animal Biology, University of Dschang, P.O.Box 67 Dschang, Cameroon; 3 Department of Animal Biology and Physiology, University of Douala, BP 24157 Douala, Cameroon

**Keywords:** Antihypertensive, diuresis, glomerular filtration rate, L-NAME, *Mammea africana*

## Abstract

**Objective:**

The present study aims at evaluating the effects of methanol/methylene chloride extract of the stem bark of *Mammea africana* on the renal function of L-NAME treated rats.

**Material and Methods:**

Normotensive male Wistar rats were divided into five groups respectively treated with distilled water, L-NAME (40 mg/kg/day), L-NAME + L-arginine (100 mg/kg/day), L-NAME + captopril (20 mg/kg/day) or L-NAME + *M. africana* extract (200 mg/kg/day) for 30 days. Systolic blood pressure was measured before and at the end of treatment. Body weight was measured at the end of each week. Urine was collected 6 and 24 h after the first administration and further on day 15 and 30 of treatment for creatinine, sodium and potassium quantification, while plasma was collected at the end of treatment for the creatinine assay. ANOVA two way followed by Bonferonni or one way followed by Tukey were used for statistical analysis.

**Results:**

*M. africana* successfully prevented the rise in blood pressure and the acute natriuresis and diuresis induced by L-NAME. When given chronically, the extract produced a sustained antinatriuretic effect, a non-significant increase in urine excretion and reduced the glomerular hyperfiltration induced by L-NAME.

**Conclusions:**

The above results suggest that the methanol/methylene chloride extract of the stem bark of *M. africana* may protect kidney against renal dysfunction and further demonstrate that its antihypertensive effect does not depend on a diuretic or natriuretic activity.

## Introduction

Since immemorial time, plants have been used in the treatment of various diseases and their importance is undeniable nowadays. Therefore, the use of medicinal plants all over the world has gained a great importance. They appear to be natural resources for new bioactive principles. So it is necessary to study the effects of those plants on various biological systems.

*Mammea africana* Sabine is a large evergreen tree that belongs to the family of Guttiferae. It grows in equatorial rain forest regions of Cameroon where it is traditionally used for treatment of stomach pains, scabies, and other skin diseases. It is also used in treatment of rheumatism pains, cough, and hypertension.[1]

The results of previous detailed studies on the chemistry of *M. africana* have revealed the presence of large number of coumarins[2,3] and xanthones typical of the Guttiferae family and a pentacyclic triterpen namely lupeol.[3,4] It has been shown that 4-*n*-propylcoumarins and 4-phenylcoumarins possess cytotoxic effects.[3] These compounds isolated from the methylene chloride fraction of the methanol/methylene chloride (1:1) extract of the stem bark of *M. africana* have shown to possess vasodilating effects.[5] It has recently been shown that this methanol/methylene chloride extract of *M. africana* is capable of preventing arterial hypertension in chronically NO blocked Wistar rats.[6] Kidney plays a central role in the control of body fluids and blood pressure and so renal dysfunction could lead to the development of arterial hypertension.[7] Conversely, renal function alteration and renal injury are important complications of arterial hypertension. It is known that long-term administration of *N^ω^*-nitro-L-arginine methyl ester (L-NAME) results in arterial hypertension, intrarenal vascular contraction, tubular and glomerular lesions and reduction in renal function.[8] Thus, L-NAME-induced renal dysfunction is a good animal model to evaluate renal protective effects of antihypertensive treatments. It has been observed that blockade of the renin-angiotensin system markedly reduces hypertension and renal alterations associated with L-NAME administration. The present study was undertaken to evaluate the effects of the methanol/methylene chloride extract of the stem bark of *M. africana* on the renal function during chronic NO synthase inhibition by L-NAME in rats.

## Material and Methods

### Preparation of Plant Extract

The stem bark of *M. africana* was collected on the bank of the Nyong River at Mbalmayo (Cameroon) in August 2002. The plant material was identified at the National Herbarium of Yaounde (Cameroon) where a voucher’s specimen number 4221/SRF/CAM was deposited. The methanol/methylene chloride (MeOH/CH_2_Cl_2_) extract was prepared by macerating 4 kg of dried ground stem bark of *M. africana* at room temperature in 10 L of MeOH/CH_2_Cl_2_ (1:1) for 24 h. The filtrate was concentrated under reduced pressure and yielded 900 g of a brown pasty extract. Five hundred milligrams of this extract were dissolved in 4% dimethylsulfoxide and the solution was adjusted with distilled water to obtain a final extract solution concentration of 20 mg/mL.

### Experimental Procedure

Experiments were performed on normotensive male Wistar rats aged 3 to 4 months and weighing 200 to 310 g. Prior authorization for the use of laboratory animals in this study was obtained from the Cameroon National Ethical Committee (Reg. N° FWA-IRD 0001954). Animals were acclimatized in individual metabolic cages for two consecutive days and blood pressure was recorded on the third day prior to the beginning of the experiment by non-invasive tail cuff using a sphygmomanometer (Panlab, 40254) coupled to a recorder (575 71 TY-Recorder). Only animals with systolic blood pressure between 110 and 130 mmHg were selected for the experiment. Twenty four hours after completion of control urine samples measurements, animals were divided into five groups of six rats each. Group 1 received distilled water and served as the control group, Group 2 received L-NAME (40 mg/kg/day), Group 3 was treated with L-NAME (40 mg/kg/day) plus L-arginine (100 mg/kg/day), Group 4 was given L-NAME (40 mg/kg/day) plus captopril (20 mg/kg/day), and Group 5 received L-NAME (40 mg/kg/day) plus *M. africana* extract (200 mg/kg/day). All treatments were administered once a day by oral route at a volume of 1 mL/100 g b.w. for 30 days. Urine samples were collected and volume was measured after 6 and 24 h at days 1, 15, and 30 following treatment and stored at –20°C for creatinine, sodium, and potassium measurements. Systolic blood pressure was once more determined at day 29. At the end of the experiment, animals were anesthetized by intraperitoneal injection of thiopental (50 mg/kg). The abdominal arteries were cannulated, blood samples were collected in heparinized tubes and centrifuged at 2500 trs/min for 15 min. Plasma samples were obtained and stored at –20°C for further measurement of creatinine. The kidneys and liver were harvested and weighed. Creatinine level was measured by means of *SGM Italia* kit and the absorbency read at 510 nm (HACH DR/2000). Na^+^ and K^+^ concentrations were measured with a flame photometer (JENWAY PFP7). Creatinine clearance was calculated and used as an estimate of glomerular filtration rate (GFR) using the following formula:

GFR (mL/min) = *U* × *V*/ (*P* × 1440)

where *U* is the urine creatinine concentration, *V* is the 24 h urine volume, *P* is the plasma creatinine concentration and 1440 is the number of minutes in a day.

### Statistical Analysis

Data are expressed as means ± SEM. Statistical significance was assessed with one-way analysis of variance followed by the Tukey multiple comparison test as a *post hoc* test using graphPad Instat Biostatistic 3.0. The difference was considered statistically significant at *P*<0.05.

## Results

### Effects of Different Treatments on Body Weight, Systolic Blood Pressure, and Organ Weight

The body weight gain in the L-NAME group was not significantly different from that of the control group. The group treated concomitantly with L-NAME and captopril had body weight loss in the first week of treatment, followed by a weak gain in weight compared to control and to L-NAME groups. In the remaining groups body weight gain was significantly low at day 28 compared to control and L-NAME groups [Table 1].

**Table 1:** Variations in body weight gain (BW gain), systolic blood pressure (SBP), kidney weight (KW), and liver weight (LW) in control animals and in the groups treated with L-NAME (40 mg/kg/day) alone or in combination with either l-arginine (100 mg/kg/day), captopril (20 mg/ kg/day), or *M. africana* (200 mg/kg/day).

*Parameters*	*Duration of treatment*	*Control*	*L-NAME*			
			*+ vehicle*	*+ l-arginine*	*+ captopril*	*+ M. africana*
BW gain(g)	Day 7	32.48 ± 2.45	20.48 ± 1.93	18.45 ± 2.34α	-0.88 ± 4.20γ^b^	20.58 ± 3.27
	Day 14	45.68 ± 2.96	42.45 ± 3.57	32.45 ± 2.09	1.35 ± 8.00γ^b^	30.53 ± 8.28
	Day 21	63.50 ± 3.02	55.36 ± 4.35	45.91 ± 3.33	18.28 ± 4.71γ^b^	49.8 ± 8.74
	Day 28	83.1 ± 2.62	72.92 ± 5.03	46.12 ± 5.61γ^a^	31.1 ± 4.33γ^b^	54.06 ± 9.81α
BW (g)	Day 30	305.65 ± 2.94	318.01 ± 5.82	292.45 ± 10.57	327.51 ± 4.63	286.34 ± 17.94^a^
SBP (mmHg)	Day 0	124.03 ± 1.87	129.31 ± 1.87	119.98 ± 1.00	125.24 ± 1.58	126.13 ± 2.44
	Day 29	130.12 ± 3.53	190.81 ± 4.18c	168.00 ± 2.03^b^β	120.90 ± 3.54γ	140.11 ± 5.24γ
KW (mg/100 g bw)	Day 30	653.07 ± 17.68	630.84 ± 16.94	779.25 ± 78.46^a^	634.99 ± 24.83	626.39 ± 16.68
LW (g/100 g Bw)	Day 30	3.59 ± 0.08	3.60 ± 0.04	3.72 ± 0.21	3.46 ± 0.10	3.44 ± 0.11

n = 6;

a *P*< 0.05

b *P*< 0.001 significantly different compared to control

α *P*< 0.05

β *P*< 0.01

γ *P*< 0.001 significantly different compared to L-NAME

The rise in blood pressure induced by L-NAME after 29 days of treatment was prevented by captopril and *M. africana*. Co-administration of L-arginine significantly reduced the level of L-NAME-induced hypertension although the blood pressure was still significantly high compared to control.

Only animals that received L-NAME concomitantly with L-arginine presented an increase in renal mass compared to control and to L-NAME-treated rats [Table 1].

### Effects of Different Treatments on the Urine Volume

**Figure 1:**
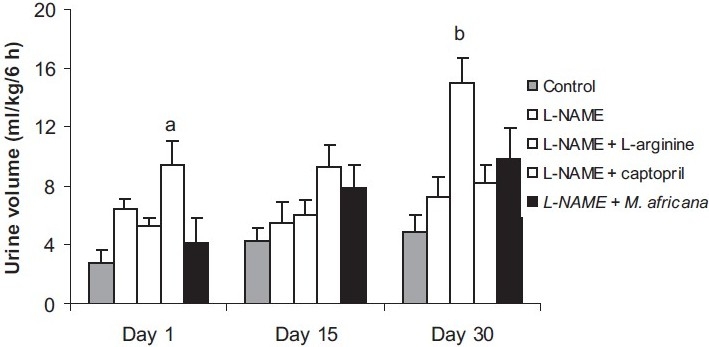
Urine volume collected 6 h after treatment in control animals and in the groups treated with L-NAME (40 mg/kg/day) alone or in combination with either L-arginine (100 mg/kg/day), captopril (20 mg/kg/day) or *M. africana* (200 mg/kg/day). n = 6; ^a^*P*< 0.05, ^b^*P*< 0.01 signifi cantly different compared to control.

**Figure 2:**
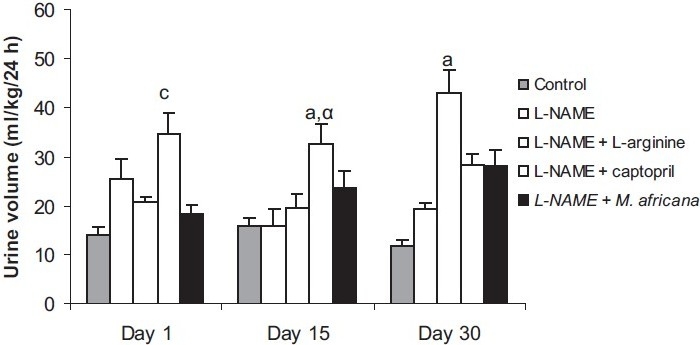
Urine volume collected 24 h in control animals and in the groups treated with L-NAME (40 mg/kg/day) alone or in combination with either L-arginine (100 mg/kg/day) captopril (20 mg/kg/day) or *Mammea africana* (200 mg/kg/day). n = 6; ^a^*P*^<^ 0.05, b*P*< 0.001 signifi cantly different compared to control; α*P*< 0.05 signifi cantly different compared to L-NAME

At the first day of treatment, L-NAME increased the urine volume both at 6 h (6.43 ± 0.74 versus 2.84 ± 0.78 mL/kg) and 24 h (25.59 ± 4.04 versus 13.99 ± 1.61 mL/kg). This effect was potentiated by captopril (9.39 ± 2.73 mL/kg at 6 h and 34.50 ± 4.47 mL/kg at 24 h) while *M. africana* extract reduced it (4.11 ± 1.67 mL/kg at 6 h, 18.19 ± 1.98 mL/kg at 24 h). When administered chronically, all the treatments caused a rise in urine volume with a significant increase in the L-NAME plus captopril group at day 15 and in L-NAME plus L-arginine at day 30 [Figures 1 and 2].

### Acute Effects of Different Treatments on the Urinary Sodium and Potassium Excretion

Acute administration of L-NAME (40 mg/kg/day) significantly increased urinary sodium excretion during the first 6 h (0.80 ± 0.13 meq/kg versus 0.27 ± 0.16 meq/kg for control) whereas no change in natriuresis was observed after 24 h compared to the control.

The potassium urinary excretion in L-NAME was similar to that of the control group after 6 and 24 h of experiment. The ratio Na^+^/K^+^ was also increased in L-NAME treated rats (*P*<0.05) 6 h after oral administration (0.71 ± 0.19 versus 0.21 ± 0.04 for control). A significant (*P*< 0.05) increase in natriuresis was also seen in L-NAME + L-arginine treated group after 24 h compared to control. No significant modification in kaliuresis was observed. L-NAME + L-arginine increased the ratio Na^+^/K^+^ (*P*< 0.01) after a 6 h collection period. L-NAME + captopril significantly reduced the ratio Na^+^/K^+^ 24 h following treatment compared to L-NAME but when comparing to the control group no modification was observed concerning natriuresis, kaliuresis and the ratio Na^+^/K^+^. L-NAME + *Mammea africana* produced a marked statistically decrease in Na^+^ excretion and in Na^+^/K^+^ ratio after 24 h compared to the control and to the L-NAME groups [Table 2].

**Table 2:** Sodium and potassium excretion in urine collected 6 or 24 h after the first treatment (day 1) in control animals and in the groups treated with L-NAME (40 mg/kg) alone or in combination with either l-arginine (100 mg/kg), captopril (20 mg/kg), or *M. africana* (200 mg/kg).

*Treatment*	*Na + (meq/kg)*		*K+ (meq/kg)*		*Na+/K+*	
	*6 hours*	*24 hours*	*6 hours*	*24 hours*	*6 hours*	*24 hours*
Control	0.28 ± 0.16	1.77 ± 0,22	1.37 ± 0.34	5.32 ± 1.11	0.20 ± 0.04	0.38 ± 0.04
L-NAME	0.80 ± 0.13^a^	2.13 ± 0.25	1.34 ± 0.19	5.74 ± 1.17	0.71 ± 0.20^a^	0.41 ± 0.04
L-NAME + arginine	0.70 ± 0.09	2.83 ± 0.34^a^	0.91 ± 0.11	6.57 ± 1.51	0.77 ± 0.04^b^	0.46 ± 0.03
L-NAME + captopril	0.42 ± 0.13	1.46 ± 0.18	0.94 ± 0.22	5.98 ± 0.45	0.36 ± 0.09	0.24 ± 0.02α
L-NAME + *M. africana*	0.27 ± 0.15α	0.41 ± 0.15^b^γ	0.95 ± 0.41	6.61 ± 0.77	0.23 ± 0.13α	0.07 ± 0.03^c^γ

n = 6

a *P*<0.05

b *P*<0.01 significantly different compared to control

c *P*<0.001 significantly different compared to control

α *P*<0.05

β *P*<0.01

γ *P*<0.001 significantly different compared to L-NAME

### Chronic Effects of Different Treatments on the Urinary Excretion of Sodium and Potassium

Urinary Na^+^ and K^+^ measured at day 15 and day 30 are summarized in Table 3. L-NAME did not affect either natriuresis or kaliuresis. As observed with acute treatment an increase in Na^+^ excretion was observed at the fifteenth (compared to L-NAME) and at the thirtieth day of treatment (compared to the control) in L-NAME + L-arginine treated group. In L-NAME + captopril as in L-NAME + *M. africana* treated rats, natriuresis and the Na^+^/K^+^ ratio significantly decreased at day 30 as compared with both control and L-NAME treated groups. But no significant change was observed in K^+^ excretion in these groups [Table 3].

**Table 3:** Sodium and potassium excretion in urine volume collected at days 15 or 30 of treatment in control animals and in the groups treated with L-NAME (40 mg/kg/day) alone or in combination with either l-arginine (100 mg/kg/day), captopril (20 mg/kg/day), or *M. africana* (200 mg/kg/day).

*Treatment*	*Na + (meq/kg/24 h)*		*K+ (meq/kg/24 h)*		*Na+/K+*	
	*15 days*	*30 days*	*15 days*	*30 days*	*15 days*	*30 days*
Control	2.20 ± 0.17	2.01 ± 0.22	7.43 ± 0.80	5.97 ± 0.55	0.31 ± 0.03	0.34 ± 0.02
L-NAME	1.67 ± 0.23	2.21 ± 0.14	5.18 ± 0.77	7.92 ± 0.86	0.33 ± 0.04	0.30 ± 0.03
L-NAME + l-arginine	3.46 ± 0.65β	3.24 ± 0.41^a^	10.59 ± 1.49	9.61 ± 1.36	0.32 ± 0.04	0.36 ± 0.06
L-NAME + captopril	1.90 ± 0.30	0.73 ± 0.11^a^β	6.68 ± 0.41	5.72 ± 0.31	0.29 ± 0.05	0.12 ± 0.02^b^α
L-NAME + *M. africana*	1.11 ± 0.25	0.26 ± 0.10^c^γ	7.01 ± 0.34	6.03 ± 0.85	0.16 ± 0.04	0.05 ± 0.02^c^β

n = 6;

a *P*<0.05

c *P*<0.01

c *P*<0.001 significantly different compared to control

α *P*<0.05

β *P*<0.01

γ *P*<0.001 significantly different compared to L-NAME

### Effects of Different Treatments on the Creatinine Level and Glomerular Filtration Rate

**Figure 3:**
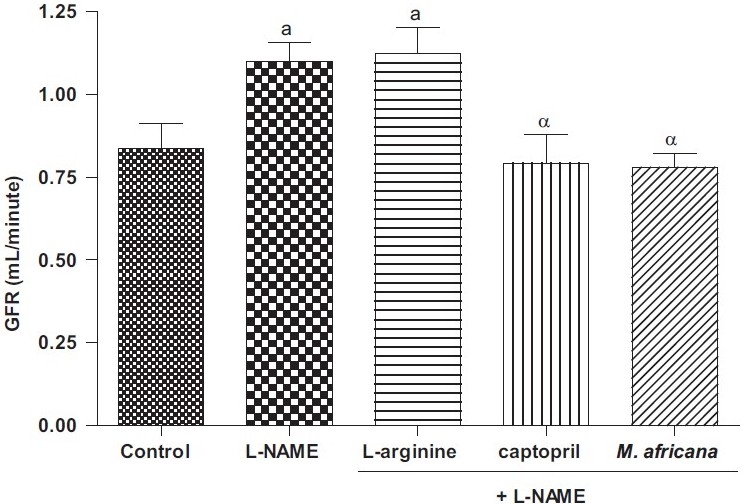
Glomerular filtration rate (GFR) after 30 days of treatment in control animals and in the groups treated with L-NAME (40 mg/kg/ day) alone or in combination with either L-arginine (100 mg/kg/day), captopril (20 mg/kg/day), or *M. africana* (200 mg/kg/day). n = 6; ^a^P< 0.05 signifi cantly different compared to control; ^α^*P*< 0.05 signifi cantly different compared to L-NAME.

The effects of chronic administration of different treatments on glomerular filtration rate (GFR) illustrated in Figure 3 showed that L-NAME given alone or in combination with L-arginine significantly increased glomerular filtration rate. Captopril and *M. africana* extract reversed the effect of L-NAME, reducing the GFR to near control values.

## Discussion

In the previous studies, it was found that *M. africana* (200 mg/kg/day) prevented arterial hypertension and left ventricular hypertrophy induced by chronic administration of L-NAME in Wistar rats.[6] In the present study we investigated the renal effects of *M. africana* in rats treated chronically with L-NAME. Results showed that L-NAME (40 mg/kg/day) induced a sustained rise in systolic blood pressure that was reversed by *M. africana* extract. In the acute treatment (24 h), L-NAME caused a transient diuresis and natriuresis that were prevented by the plant extract. In the chronic treatment, the extract induced a potent anti-natriuretic effect with a non-significant increase in diuresis.

Both *M. africana* extract and captopril were able to completely reverse the arterial hypertension induced by L-NAME. These observations corroborate previous results[6,9] and further confirm that *M. africana* extract and angiotensin-converting enzyme inhibitor are convenient substances for the management of arterial hypertension in NO-deficient subjects.

In acute treatment, L-NAME increased diuresis and natriuresis which were significantly reduced by the *M. africana* extract. These results are in agreement with those of Qiu *et al*.[10]; Baylis *et al*.,[11] and Qiu *et al*.[12] who found marked natriuretic and diuretic effects after acute nitric oxide synthase inhibition by L-NAME. It is known that an increase in arterial blood pressure leads to an increase in renal perfusion pressure and further to natriuresis and diuresis through the stimulation of intrarenal baroreceptors. It has been shown that *M. africana* extract possesses vasorelaxant effects.[5] These vasorelaxant effects may contribute to the reduction in renal perfusion pressure, thus reducing the natriuretic and the diuretic effects of L-NAME. In the chronic treatment, captopril and *M. africana* extract induced a marked reduction in natriuresis even though a slight increase was observed in urinary output as compared to control and L-NAME groups. This chronic effect may also result from their blood pressure-lowering effects. It is thought that the renal function regulation is mainly exerted by nitric oxide.[9,13,14] In fact, NO exerts direct inhibitory effects on renal tubular function through the inhibition of both the Na^+^/K^+^-ATPase and the Na^+^/H^+^ exchanger,[15,16] therefore, reducing sodium reabsorption and increasing natriuresis. Thus, the inhibition of NO production will conversely results in sodium retention. Moreover, it is well documented that chronic L-NAME treatment increases the expression of Na^+^/K^+^-ATPase, Na^+^/H^+^ exchanger and the Na^+^/K^+^/2Cl^-^ co-transporter[16] that contribute to increase sodium reabsorption. In the present study a non-significant reduction in sodium excretion was observed in 15 days L-NAME-treated animals. This reduction was potentiated by *M. africana* extract inferring that this extract neither acts as nitric oxide synthase stimulator nor as a nitric oxide donor which is known to selectively inhibit an apical Na^+^/K^+^/2Cl^-^ co-transpoter.[17,18] These results are in accordance with our previous finding where we showed that the extract has a potent antihypertensive activity but could not ameliorate the endothelium-dependent vasorelaxation in L-NAME-hypertensive animals.[6]

During the acute treatment, L-NAME significantly increased the ratio Na^+^/K^+^ that was significantly reduced by plant extract. In chronic treatment, the ratio was not affected in L-NAME-treated animals, but significantly reduced in the group treated with L-NAME plus *M. africana* extract. The effect of this extract is similar to that of mineralocorticoids such as aldosterone which increase nephron sodium reabsorption and therefore reduce the Na^+^/K^+^ ratio.[19,20] This effect is in contradiction with its antihypertensive property and demonstrates that the antihypertensive effect of *M. africana* extract is not mediated through diuretic or natriuretic effects. As a plant extract is a mixture of many compounds, the substance responsible for the antidiuretic and antinatriuretic effects may be different from the one responsible for the antihypertensive effect.

The gain in body weight was significantly reduced in extract and captopril-treated groups. A similar result was obtained by Nguelefack-Mbuyo *et al*.[6] and Afkir and co-workers[21] using another angiotensin-converting enzyme, enalapril. The exact mechanism of this effect is yet to be known.

The glomerular filtration rate was significantly increased in L-NAME and in L-NAME plus L-arginine-treated Wistar rats. Results concerning the effects of L-NAME on glomerular filtration are conflicting. Some researchers found a decreased glomerular filtration rate during NOS inhibition,[9–12] whereas others observed no change in glomerular filtration rate after L-NAME treatment.[22,23] Van Dokkum and co-workers[24] observed an increased glomerular filtration rate in L-NAME uninephrectomized heterozygous fawn-hooded hypertensivexAugust Copenhagen Irish (FHHxACI) F1. These rats developed focal glomerulosclerosis, which was caused by a larger hemodynamic burden as a result of hyperfiltration as explained by the authors. Thus, it could be assumed that animals treated here with L-NAME developed renal damage that was prevented by captopril and *M. africana* extract.

In conclusion, the present study demonstrated that the methanol/methylene chloride stem bark extract of *M. africana* prevented the hypertension induced by chronic administration of L-NAME. In addition, the same extract exhibited an antidiuretic effect and protected the kidney from increased glomerular filtration rate. The above results suggest that the cardiovascular effects of *M. africana* may be due to its vasodilatory properties or possibly by interfering with the angiotensin II pathway as its effects resemble those of captopril.
